# Willingness of pregnant and postpartum women who use marijuana and/or cannabidiol to participate with their offspring in long-term cohort studies: an exploratory study

**DOI:** 10.3389/fpsyt.2025.1641467

**Published:** 2025-10-21

**Authors:** Deepthi S. Varma, Amie J. Goodin, Bruce A. Goldberger, Kay Roussos-Ross

**Affiliations:** ^1^ Department of Epidemiology, College of Public Health and Health Professions, University of Florida College of Medicine, Gainesville, FL, United States; ^2^ Pharmaceutical Outcomes and Policy, Center for Drug Evaluation and Safety (CoDES) Consortium for Medical Marijuana Clinical Outcomes Research, University of Florida, Gainesville, FL, United States; ^3^ Department of Pathology, Immunology and Laboratory Medicine, University of Florida College of Medicine, Gainesville, FL, United States; ^4^ Department of Obstetrics and Gynecology and Psychiatry, University of Florida College of Medicine, Gainesville, FL, United States

**Keywords:** marijuana, cannabinoids, perinatal, long-term cohort studies, participation

## Abstract

**Background:**

Prevalence of marijuana and cannabinoid use is increasing among reproductive-age women. There are uncertainties regarding long-term impacts of marijuana and/or cannabinoid exposure among pregnant women and their offspring. Longitudinal cohort studies of marijuana and/or cannabinoid exposed mother-infant dyads is the best way to ascertain the long-term impacts. However, previous studies have shown enrollment, and long-term retention are challenging in substance-exposed women.

**Objectives:**

This study explores the willingness of pregnant and postpartum women who use marijuana and/or cannabidiol to participate with their offspring in long-term cohort studies.

**Methods:**

We conducted 4 focus group discussions and one individual one-on-one interview with a total of 17 pregnant or postpartum women using an IRB approved interview guide. All interviews were audiotaped, transcribed and analyzed using the computer assisted qualitative data analysis software Atlas ti™. We used a deductive content analysis approach and utilized consensus coding procedures.

**Results:**

Marijuana and/or cannabinoid-exposed pregnant women are willing to participate in long-term research studies with their babies if they can build a trusting relationship with the research staff and are confident of their anonymity, as protection from negative consequences was a key concern. They would also like to understand in detail what type of data are collected, when and who all will see it and what will be done with the data before they provide the consent. All participants agreed that incentives are important and had various suggestions regarding the type and frequency of incentivization.

**Conclusion:**

The concerns and needs of marijuana and/or cannabinoid-exposed pregnant women recruited for research should be considered carefully in designing study protocols.

## 1 Introduction

Recent prevalence estimates of marijuana (cannabis) use during pregnancy ranges from 2 to 36%, with higher rates seen in young women, urban centers, and when assessing use based on toxicology compared to self-report (1). Cannabidiol (CBD) use in the general population is also increasing, due to the public perception that it is safer than the psychoactive cannabis component Δ9-tetrahydrocannabinol (THC) (2), though it is unclear whether CBD use during pregnancy is increasing as current literature documenting CBD use patterns in pregnancy is lacking. The mechanisms driving cannabis-induced pregnancy complications are still unclear and findings on studies exploring the relationship between prenatal cannabis use and offspring outcomes are mixed.

A recent study on prenatal cannabis use and maternal pregnancy outcomes reported that the prenatal cannabis use was associated with greater risk of gestational hypertension (aRR, 1.17; 95% CI, 1.13-1.21), preeclampsia (aRR, 1.08; 95% CI, 1.01-1.15), gestational weight gain (GWG) less than (aRR, 1.05; 95% CI, 1.01-1.08) the guidelines and greater than (aRR, 1.09; 95% CI, 1.08-1.10) the guidelines, and placental abruption (aRR, 1.19; 95% CI, 1.05-1.36) (3). A recent study by the same group that investigated maternal cannabis use during early pregnancy and its association with offspring attention deficit hyperactivity disorder (ADHD) and disruptive behavior disorders (DBD) found no association (4). Findings from a meta-analysis on cannabis exposure and the risk for neuropsychiatric anomalies in the offspring reported mixed results (5). For example, based on the 17 studies that were included in the final quantitative analysis (n=534,445 participants), prenatal cannabis exposure was not associated with an increased risk of autism spectrum disorders (ASD), psychotic symptoms, anxiety, or depression in offspring. However, they reported that it may slightly elevate the risk of ADHD and predispose offspring to cannabis consumption (5).

Recent studies have shown that the perceived impact of legalization of marijuana by different states have resulted in easier access (via retailers and delivery), greater acceptance (including reduced stigma and more discussions about prenatal cannabis use with health care practitioners), and trust in cannabis retailers (including safety and effectiveness of diverse products sold and perceptions of cannabis retailer employees as knowledgeable, nonjudgmental, and caring) among all populations including pregnant women (6–10). Pregnant women who use THC/CBD could potentially face legal challenges that makes them weary of accessing health care for themselves or their newborns. Though there are variations across States regarding the reporting of substance use status of pregnant and postpartum women, most women who use substances are not clear about these legalities and hence fear a visit by the Department of Child and Family (DCF) or comparable agency and losing the custody of their newborn. All states incorporate some reporting requirement into their statutes, regulations, or policies; for instance, some states statutorily define child abuse or neglect to include the birth of a substance-exposed or -affected newborn (11). Additionally, fear of stigma, medical conditions or socioeconomic status all resulting from continued substance use during pregnancy and postpartum also prevent these women from participating in any health research or accessing any type of health care.

Intrauterine environment and perinatal exposures play a crucial role in the development of the fetus and influence the development of adult-onset disorders (12). Due to legal, ethical and practical challenges many studies assess prenatal exposures to harmful substances such as opioid or marijuana via retrospective recall which is prone to recall bias. Research has shown that contemporaneous assessment of the prenatal exposures to harmful substances by initiating recruitment during pregnancy is preferrable to collect more accurate information to assess its impact on the mother-infant dyad (13). However, longitudinal cohort studies of pregnant women who use THC/CBD and their offspring face notable methodological complexities to design and implement.

The objective of this qualitative study is to explore the barriers to recruitment and suggestions for retention of pregnant women who use THC/CBD and their offspring for a 5-year longitudinal cohort study that involves periodic developmental assessments (of the offspring) and biological sample and imaging collection from both the mother and the offspring.

## 2 Materials and methods

### 2.1 Participant recruitment

A total of four focus group discussions (FGDs) and one individual one-on-one interview was conducted from March to June 2023 with a total of 17 women who were either pregnant or postpartum. We had 2,3,5 and 6 participants respectively in each FGDs. Participants were recruited from women’s health clinics, and an inpatient substance use treatment program for pregnant and/or postpartum women within the same town. IRB-approved study flyers were mailed to the inpatient treatment program that specifically works with pregnant and parenting women with substance use disorders in the community and posted in various community locations and women’s health clinics. Women who contacted us through the phone number provided on the recruitment flyers were assessed for their eligibility and were given several date and time options for the FGDs. Two FGDs and the one-on-one interview were conducted online via Zoom and two other FGDs were conducted in person at the inpatient treatment center. All participants provided signed informed consent, completed a sociodemographic questionnaire before participation in the FGD, and received compensation of $30 US for their time and effort. All women enrolled in FGDs were at least 18 years old, currently pregnant, breastfeeding, or caring for a child who was less than 5 years old and reported current or past lifetime use of any marijuana product or any product containing CBD (e.g., vapes, smoking, tinctures, oils, ointments, or any type of edible marijuana or CBD-containing product). This study was approved by the University of Florida Institutional Review Board (IRB 202201895).

### 2.2 Interview guide development

The FGD data was collected using a semi structured interview guide developed based on the framework informed by Kreuger & Casey (2020) (14). The focus of the interview guide was to understand the willingness and feasibility of recruiting and retaining pregnant and postpartum women for a longitudinal research study on the long-term outcomes of cannabis and CBD use during pregnancy. The semi structured interview guide was developed after extensive literature review and periodic review and inputs from experts in the field.

### 2.3 Data collection process

All FGDs were conducted by the first author (DV) who is a qualitative research expert along with one other team member who served as the note taker. Out of the four FGDs, two were conducted online via Zoom while the other two were in person at a location decided based on mutual convenience, privacy and participant safety. The one in-depth interview was also conducted online via Zoom. The FGDs and the one-on-one in-depth interview used the same interview guide and all interviews lasted for a maximum of 90 minutes, were audio recorded with consent, and transcribed verbatim for analysis. All participants received a $30 US gift card at the end of the FGD or the interview.

### 2.4 Data analysis

Data was analyzed using Atlas ti™ version 23.2.1the qualitative data analysis software (15). The transcribed data was uploaded to Atlas ti™. We used a deductive content analysis approach to analyze the data. Two coders (DV and a graduate student level coder) started coding independently each of the FGD transcript using an *a priori* coding list that was generated based on the interview guide and in consultation with the research team (**Table 1**). During the first cycle of coding new codes were added to this list as we proceeded with the coding of each transcript. We determined that data saturation had been reached when no new codes emerged from the transcripts during our coding process of the fourth transcript, leading us to halt further focus group discussions after the fifth session. During the second cycle, codes that reflect the same topic or are similar were grouped under a theme. **Table 2** provides a list of themes, codes and their definitions.

**Table 1:** List of apriori codes based on the interview guide*.

List	Questions	Codes
1	Willingness to enroll in a study	Yes, No
2	Reasons to enroll	Trust, to help others, want to know more, attractive study features
3	Source of invite	Doctor, researcher, other
3	Personal information collection impacting research participation	No impact/Yes impact
5	Interview format preference, reasons	In-person/online/face-to-face via zoom
6	Survey format preference, reasons	In-person/online/face-to-face via zoom
7	Sharing sensitive information preference, reasons	In-person/online/face-to-face via zoom
8	Substance use information preference, reasons	In-person/online/face-to-face via zoom
9	Concerns about medical records sharing	Fear of misuse, quantity,
10	Concerns about urine drug screening	Willing, unwilling, types of concerns
11	Concerns about meconium screening	Willing, unwilling, types of concerns
12	Concerns about cord blood screening	Willing, unwilling, types of concerns
13	Concerns about MRI screening of infants	Safety concerns, other concerns
14	Concerns about providing breast milk samples	Willing, unwilling, types of concerns
15	Concerns about periodic developmental assessments of their child	Willing, unwilling, types of concerns
16	Willingness to allow collecting sensitive information from their children	Domestic violence, sexual abuse, other sensitive information
17	Preferences regarding incentives, reasons	Type, quality, quantity
18	Home visits preferences, reasons	Privacy, intrusive, convenient, depends on several factors
19	Preferences on scheduling follow up visits, reasons	Advance scheduling, reminders, align with other hospital follow up appointments

*These codes were developed initially from the interview guide prior to transcript coding and were subsequently refined as the coding process progressed.

**Table 2:** Relevant themes and codes from the focus group discussions.

Relevant Themes and Codes from the Focus Group Discussions			
	Themes	Codes	Definitions
1	Factors influencing willingness to enroll in a 5-year study	a) Reasons to enroll	Participants discuss the various reasons why they would be willing to enroll in a long-term study along with their newborns
		b) Source of invite to join the study	Participants discuss the significance of the source of invitation to participate in a study and its impact on enrollment
		c) Impact of personal information collection on enrollment	Participants discuss the impact of their awareness of various types of personal information that would be collected during the study on enrollment
11	Preferences for reporting on CBD/marijuana use	a) On personal and sensitive information	Participants discuss the barriers and facilitators to providing personal and sensitive information
		b) On CBD/marijuana use	Participants discuss the barriers and facilitators to providing information regarding their CBD/marijuana use to researchers
		c) Consent to review medical records	Participants discuss the barriers and facilitators to providing consent to review medic al records to researchers
		d) Consent to collect biological samples	Participants discuss the barriers and facilitators to providing consent to collect various types of biological samples for the research study
		e) Consent to do periodical MRI during pregnancy or later for the child	Participants discuss the barriers and facilitators to providing consent to do periodical MRI during pregnancy or later for the child
		f) Consent to conduct periodical developmental assessment of the child	Participants discuss the barriers and facilitators to providing Consent to conduct periodical developmental assessment of the child
III	Facilitators for retention in the study	a) Incentives	Participants discuss the role of incentives as a facilitator to research enrollment and retention
		b) Home visits	Participants discuss how home visits by the researchers for study assessments could serve as a facilitator to research enrollment and retention
		c) Scheduling the study follow up visits	Participants discuss the role of scheduling the study follow-ups aligning with their regular follow up visits could act as a facilitator to research enrollment and retention

## 3 Results

### 3.1 Demographic characteristics

We had a total of 17 participants in the FGDs. The mean age of the sample was 32.9 (range 22-47; SD 5.3). We had 1 participant identify as African American race and 1 participant identify as Hispanic ethnicity. All other participants identified as White race (n=16), and non-Hispanic ethnicity (n=16). All participants (n=17) had Medicaid as their health insurance. 2 participants had a Doctoral or professional degree, 1 had a Master’s degree, 7 had a Bachelor’s degree or some college, 6 had completed high school, and another 6 reported not completing high school. 7 (31.8%) participants were currently pregnant and 4 (18.2%) were breastfeeding or pumping for their newborns.

### 3.2 Qualitative data results

The qualitative data analysis focused on exploring the willingness to enroll in a long-term cohort study along with newborn babies and understanding the factors that act as barriers and facilitators to different types of data collection including biological samples and retention in the study. Findings are presented under three major themes (see **Table 1**): 1) factors influencing willingness to enroll in a 5-year study, 2) facilitators and barriers to data collection, and 3) facilitators for retention in the study. Exemplar quotes are presented below. Additional quotes are given in **Table 1** as the **Supplementary Materials**.

#### 3.2.1 Factors influencing willingness to enroll in a 5- year study

##### 3.2.1.1 Reasons to enroll

Sixteen women were willing to enroll in a five-year study along with their newborns because they thought it was important, or they wanted to receive information regarding the effects of marijuana and/or CBD on themselves. This motivation may stem from a genuine interest in the scientific community’s understanding of these substances, as well as a personal desire to gain insight into their potential impact on their own health and well-being.

I would like to be involved in something like this because I would like to get information about my child or information on this stuff (CBD use).(P16)

Some women specified certain conditions for participating in the study, including incentives, ensuring their child’s anonymity, flexible scheduling, and the option for advance appointments or integration with their prenatal care visits.

You know, as long as I knew far enough in advance when the appointments would be because my schedule does book up. So, I’d be willing to come, even if it was outside of the times, just as long as I…(P01)

##### 3.2.1.2 Source of invitation to join the study

Fourteen women mentioned that the source of invitation to participate in the study did not matter as long as they were provided with all the required information regarding the study. Some women however mentioned the importance of keeping their names anonymous by the researchers.

It wouldn’t matter who the invitation came from, as long the P.I. of the study was listed, and the link, maybe … to their bio (was given) … so that I could look into what they research … And, you know that they’re a reputable scientist (P01)

Some women mentioned that they were not comfortable enrolling in a study after seeing the study advertisement flyers alone. They preferred being referred to the study by a professional or being contacted by a professional specifically because this is a study on cannabis use.

I agree that (the flyers) at the bus stop, or the hotel, or the side of the road … isn’t it like a scam or something?… Since it’s about weed, somebody that looks professional or is coming from a hospital, or somebody that I would actually trust is better (P14)

##### 3.2.1.3 Impact of personal information collection on enrollment

Most participants mentioned that knowing the researchers would collect personal information would not impact their decision to enroll as long as they were sure of the anonymity of the data reported. However, some women mentioned that their decision to participate might be impacted after learning of what type of personal information the researcher would collect.

You know … the information be coded and presentations and things like that, if any pictures and things like that is used, that’s discussed in advance. But, as long as it’s not shared (outside) …that I’d been coded and my child had been coded for statistics, giving to the statistics team, or giving to the outside collaborators or things like that….I am okay (P01)

Two participants mentioned that if they were clearly informed of all the information collected for the study in advance they wouldn’t be offended later when those questions were asked by the researcher.

I feel like once I’m informed of what the study is about and what all is required of the study and the level of questioning, that’s all part of the initial talk? I wouldn’t be offended by any personal questions about my life …(P15)

#### 3.2.2 Preferences for reporting on CBD/marijuana use

##### 3.2.2.1 On personal and sensitive information

Almost all participants were comfortable with either zoom call or in-person as method of data collection. One participant specifically mentioned that since personal and sensitive information would be collected, they preferred an in-person interview.

I would prefer in person if it’s dealing with more personal information, and it will be deeper I would just prefer in-person…(P13)

##### 3.2.2.2 On CBD/marijuana use

Participants had several different opinions about how they would like to report the CBD/marijuana use to the researchers. Some participants mentioned that they could report via text messages or phone calls. Another participant mentioned using logs or dairies to note use and then provide that to the researcher when needed.

I mean, I think it would make things easier if it doesn’t have to be reported every time I consume. Like with asthma patients, you have a log, right? And you just record there for a month. So, maybe a monthly recording could be enough, something you do at home. And then you just give that information to the person in charge….(P04)

Two participants mentioned the reporting method would depend on the type of information being collected, the timing of collection, and the context in which the reporting occurs.

I don’t think it really matters to me necessarily which way. It would depend on when I was gonna report if I was in here (the rehabilitation center) or if I was home … in person, on the phone, text, whatever…. either way is fine with me. However,’ s convenient at that time, it doesn’t matter…(P11)

##### 3.2.2.3 Consent to review participant’s medical records

Participants had varied responses to the question of providing consent to allow researchers to see participant’s medical records as part of the research study. Many participants mentioned that they want the researchers to be professional and inform them about exactly what information the researchers will look at and collect from their medical records before they agreed to consent. Additionally, one participant mentioned that some women have legal concerns regarding non-medical marijuana use in a state where recreational use is not legal yet.

In my opinion, the identification process needs to be thoroughly described … maybe even giving an option “Okay, this is the type of data we’re going to collect. Is there anything that you’re not comfortable with us collecting that you would like us not to collect?” and giving them that option? (P01)

I feel that personally, and maybe for other women too, the concern would be the legality of the situation. Our state is medical (marijuana) only, so for those who are not using medical, it may be concerning to them as to how the information is handled, and what kind of repercussions they could possibly face due to sharing that information. (P05)

Only two participants mentioned that they will not give consent for the researchers to look at their medical records.

I don’t, I don’t think that my answer would be yes, because when you have, when you give someone access to all of your medical records that is literally like *all* of your medical records— I don’t think I would say yes just because, like, there’s a certain amount of like, like privacy, like, for my life and livelihood that I feel like, I wouldn’t want to give someone information—to all of something that was so personal to me.(P12)

One participant mentioned that she preferred self-report instead of medical record access. She specifically mentioned her fear of her information being accessed by the State or Department of Child and Family (DCF).

I would rather just report that. I feel like that (medical records) would connect our name too much to the number maybe…. where would the information be stored? Would any of it be given to any state, like to the DCF? (P11)

##### 3.2.2.4 Consent to collect biological samples (urine, meconium, cord blood, breastmilk)

Participants had mixed opinions regarding providing consent for biological samples. Some women believed that the hospitals already request them for their consent to collect and conduct drug screen test on their urine and meconium samples during delivery.

They (hospital staff) did it with my baby. When you are signing all the documents, that’s part of the documents that they’re signing. To agree to do a drug screen…(P12)

While some other participants were fearful of legal repercussions regarding a false positive or an actual positive drug screen test result. Participants reported similar beliefs and concerns regarding the meconium sample collection too.

I think the legalities of the situation may deter people from being willing to do those routine drug tests. It would definitely be a big concern for me personally … It can cause more harm than good, I suppose, in many ways. (P05)

All participants, except one, were willing to provide consent to donate a sample of their cord blood unless someone desired to bank their cord blood for later personal use. The one participant who was unwilling was fearful whether the researchers will use the cord blood for something else other than research purposes.

If i’m not banking, and it’s okay that I can do delayed clamping, then I would be comfortable giving blood from that. (P01)

In the case of breastmilk, the main concern was the amount requested from the woman for research purposes. Most women were willing to provide a small amount, up to 15ml, for research purpose.

A minimum amount like 15 mils wouldn’t be that hard for somebody to produce (and give) if you’re using it for the purpose of drug testing … you really don’t need much more than that…(P03)

One participant mentioned that she would like to know exactly what the researchers would examine in the breastmilk before consenting to give a sample.

I would want to know what you’re looking for and what exactly is targeted in the breastmilk before I consented. (P7)

##### 3.2.2.5 Consent to do periodical MRI during pregnancy or later for the child

Participants were generally unsure about exposing themselves to MRI while they were pregnant. Many wanted to know the effect of MRIs or any radiation from MRI on their unborn child. Some women were also concerned about the reasons researchers were asking for a MRI in pregnancy feeling that MRIs are avoided in pregnancy.

Unsure. I need to do some research on how that would influence the baby as far as radiation exposure. Yeah, and like all the effects of what could happen … positive and negative, and their experience and like what happened to those who did before me. I am extremely interested in the study if it is safe for my baby, I would like to see more research before I consent (P11)

In the case of newborn MRI, some participants were willing to consent for newborn MRI as long as they were allowed to observe the whole process. Some women said they were willing to consent since they want to do *“whatever it takes”* to make sure that their babies are doing well.

I was gonna say that I’d have no problem. I’d want the option of participating in that and the option of coming with my child, standing in the observation room, know the results of it, and at least there is something I need to be concerned about. And I’d also want to know if there are any specific risks to my child participating in that. If there’s any kind of exposure that could affect them later. I mean, I wouldn’t want to expose them to something since they (babies) are brand new (P03)

Some participants were not comfortable giving consent citing fear of exposing their babies to radiation.

I’m not comfortable with my child being exposed for unnecessary reasons. (P7)

##### 3.2.2.6 Consent to conduct periodical developmental assessment of the child

Most participants were willing to give consent for periodic developmental assessments of their child. However, several participants were either unsure or declined consent to collect information from their children regarding domestic violence or sexual abuse.

There is no way that I would let someone talk to my child about sexual abuse when they’re not being abused. That’s crazy. I wouldn’t open up for a person to come in and talk … my children will never know anything about sexual abuse until they’re older … how are you gonna come in and ask my 4-year-old son if he’s being sexually abused? That’s really messed up…(P12)

Two participants expressed concern that no mother would willingly give consent for researchers to investigate allegations of abuse or neglect within their home environment.

But if that (abuse or neglect), is happening in the house, I doubt that any mom is going to accept that happened, so you are never gonna know … but those kinds of people maybe don’t … are not even interested in being in a study…(P04)

Three participants shared their experiences of childhood sexual abuse, which motivated them to educate their own children about the issue. As a result, they were more open to their children being asked sensitive questions by researchers in their presence.

I was raped almost my whole life since 6 months old by my dad, and a cop, and other cops were nice to me….now I would not let y’all … or I would not send my child into a room with somebody I would have to be there….(P16)

Three participants were willing to give consent to ask their children about domestic violence or any sexual abuse as long as the researchers made reporting requirements clear to the parents ahead of time.

I’m fine sharing those type of things (sexual abuse). Like I’ve stipulated before, you know, making sure it’s very clear what your required reporting is….(P01)

#### 3.2.3 Facilitators to enrollment and retention in the study

##### 3.2.3.1 Incentives

One participant suggested that the type and quantity of incentives should be adjusted according to the specific requirements of each follow-up task. The participants also proposed tailoring the compensation to meet the unique needs of everyone. One participant provided a specific example, expressing a preference for a visa card, that could be used anywhere, as opposed to a gift card to a specific location. Another participant felt that it would be beneficial to provide incentives that cater to each participant’s individual needs. Other suggestions made by the participants included the provision of a lactation consultant to assist with breastfeeding issues, toys for the children and different types of incentives for completing different tasks throughout the study period.

I feel you could maybe try to tailor it specifically for people’s needs. If somebody needs childcare you could offer them childcare, if somebody needs money, you could offer that, or transportation, or, whatever they might need…. Or, you know, like things, toys? Money is a big thing, and then obviously toys, anything bright and shiny…(P13)

As far as compensation goes, I think a gift card is acceptable. I think if not a gift card, maybe even like a reloadable debit card. (P05)

##### 3.2.3.2 Home visits

Several participants mentioned that regular home visits by the researchers to conduct the assessments, rather than the participants to travel to the research site would be a facilitating factor to stay engaged in a long-term study. However, three participants mentioned that the decision to agree to a home visit would depend upon their condition at that moment.

I would, like, prefer it if you guys came to us, because it makes things easier with you know, especially because I have two children, I would prefer that … also depending on my ride. (P12))

I feel like that’s the best choice, especially for moms who don’t have any transportation or access to transportation. Also, I feel like the child’s more likely to be open and honest in the comfort of their own home.(P13)

##### 3.2.3.3 Scheduling the study follow up visits

Another facilitating factor mentioned by the participants was to consolidate all research-related tests into a single day, either during their hospital stay or during a scheduled follow-up appointment with their doctor. This would enable the participants to complete all necessary tests in a convenient and efficient manner. Several participants emphasized the importance of advanced scheduling for the study visit, suggesting that it would greatly simplify the process and make it more manageable. One participant specifically noted that having a schedule in place six months prior to the actual appointment, with reminders sent two weeks in advance, would be highly beneficial in ensuring that they are well-prepared and able to attend the study visit.

So, I think, having a consideration of when, during the stay it is performed would be an important factor. And then trying to align it potentially with when the baby needs blood work or other things… (P01)

## 4 Discussion

Most participants in this study were willing to enroll and complete a longitudinal 5-year study along with their newborns and were clear about who should invite them to participate in the study (16). Participants were not comfortable responding to advertisements or self-enrolling in a study through a recruitment flyer due to fear of being scammed. Previous studies have shown that trust in the recruiter and the institution conducting the research is a crucial factor in recruitment success, especially among hard-to-reach or vulnerable populations (16, 17). Further, passive recruitment strategies such as flyers have been found to be less successful in motivating interested participants to take the extra step to respond to a flyer by calling, texting, emailing or even face-to-face recruitment by the research staff (18–21).

Next, our findings reinforce previous observations reporting pregnant women’s willingness to participate in research because they believe it is crucial and may provide them with valuable information that could benefit both themselves and their children (22, 23). Several studies have highlighted the uncertainty surrounding the potential adverse consequences of perinatal marijuana use, as well as the limited information and lack of discussion with healthcare clinicians regarding the impact of perinatal marijuana use on birth outcomes (24, 25). This is particularly significant, given that all participants in our study had a history of past or current THC/CBD use. The willingness of these mothers to participate in the study suggests that they are proactive, engaged, and invested in their own health and the health of their children, and are willing to contribute to the advancement of scientific knowledge to promote the well-being of themselves and their families.

Other factors such as incentives, anonymity especially of their child and flexible schedule mentioned by our participants have also been reported as facilitators for enrollment and retention in longitudinal studies previously (16, 26). Flexible schedules including researcher’s willingness to adjust study appointments according to participant convenience, aligning with other clinic appointments was emphasized while discussing facilitators to enrollment and continuation in a longitudinal study by several participants. This is also related to the woman’s inability to make multiple trips due to lack of transportation, childcare issues or inability to get off their work frequently. Anonymity was mentioned by many as especially important since many of these women constantly live under shame of using substances during pregnancy and the fear of being reported and losing the custody of their newborn for using THC/CBD during their pregnancy (16, 27–29). These fears are augmented by the opposing state laws surrounding pregnancy and substance use, with some states prioritizing access to treatment for pregnant individuals struggling with addiction, while others categorize substance use in pregnancy as child abuse or impose severe consequences through civil or criminal sanctions (30). Specifically, fear of loss of anonymity and the potential for the researcher to access comprehensive participant medical histories hinders informed consent in several cases. This phenomenon may be attributed to the lingering effects of punitive policies and societal stigma surrounding maternal substance use, which participants have either personally experienced or witnessed (27, 29). These policies, which often perpetuate negative attitudes towards substance use during pregnancy and postpartum, have created a culture of fear and mistrust among participants, influencing their willingness to disclose information and engage with research related to this topic. Conversely, participants who did consent emphasized the importance of trust and a positive relationship with the researcher in facilitating informed decision-making. Prior research in this area also has emphasized the key role of trust and rapport with the researcher as crucial in getting consent to participate and facilitate different types of data collection (16, 17, 31).

Participants in this study demonstrated varying levels of willingness to donate biological samples for research studies. As reported in one of the recent studies our participants also mentioned that the hospitals already collect urine and meconium (16). The hesitation to provide colostrum or breastmilk could be due to the woman’s concerns about having adequate breastmilk to feed their babies. Providing them with a clear understanding of the quantity that would be collected by demonstrating the sample collection process could help alleviate their concerns about losing too much. Periodic MRI during pregnancy and of the newborn was another topic that generated mixed response from participants. Some participants mentioned safety of these imaging modalities as their key concern. Despite the valuable diagnostic information provided by medical imaging modalities, there continues to be concerns among people regarding the risks related to repeated radiation exposure to both maternal and fetal health (32). These concerns prevent many pregnant women from providing consent for neonatal MRI or MRI during pregnancy (33–35). Further, the findings suggest that among women who agreed to provide consent for MRI during pregnancy or for their newborn, a strong desire for reassurance and safety emerged as a primary motivator. Specifically, many of these women expressed a deep-seated need to be present and observe the imaging process to ensure their baby’s safety. This behavior appears to be rooted in a complex interplay of factors, including a mistrust towards researchers in general, history of substance use, concerns about fetal or newborn’s health due to their substance use history, and a heightened sense of maternal responsibility (34). A detailed information session with the woman about the fetal MRI procedure, containing clear-cut explanations about the purpose, course, method and possible distressing conditions would be helpful in alleviating the woman’s anxiety and in facilitating consent (33).

Incentives, particularly financial ones, have consistently been identified as a key facilitator in the recruitment and retention of research participants (16, 31, 36–39) In this population, incentives in the form of gifts for their babies were especially appealing, as financial hardships related to substance use often limit their ability to purchase such items (16). As in earlier studies our participants also suggested that since women who use substances may have varied needs it would be useful if the researchers could tailor the incentives based on the need of each participant (40, 41). Another important point raised was the alignment of the research study visits with their medical clinic visits so that they didn’t need to travel twice or arrange for childcare or transportation twice. Previous studies have shown that many women who use substances may not have transportation of their own or may be restricted from driving due to loss of licensure making it difficult for them to come for follow ups or research appointments (16, 32). Further, they may not have the support system of financial capability for childcare making it difficult for them to travel for appointments multiple times. This was also highlighted by participants sharing a preference for home visits by research staff for periodic assessments of their children. However, there were a few participants who did not want anyone to visit their home and preferred to come to the research study location for assessment. This could be due to their fear of being judged by the staff on how or where they live which also if reported to authorities could lead to punitive measures.

## 5 Conclusion

Findings from this study suggests that pregnant or postpartum women who use THC/CBD are willing to participate in long-term studies along with their newborns but continue to be fearful of being reported and losing the custody of their child. Trust towards the researcher built through a strong rapport is crucial for successful enrollment and retention of this population in long-term research studies. The traditional model of obtaining consent and collecting different types of data may need to be adapted to accommodate the unique needs and concerns of pregnant and postpartum women who use THC/CBD. A more effective approach might involve integrating emotional and psychological support, while also adopting a flexible framework that allows for adaptations and adjustments as needed.

### 5.1 Strengths and limitations

This study is the first of its kind that explores the willingness of pregnant and postpartum women with a history of marijuana and or cannabidiol use to participate in a 5-year long longitudinal study that includes mother-infant dyads. Previous studies have focused on women who use opioid and not specifically THC/CBD use.

One of the limitations of this study is that all participants are from a single state where recreational marijuana use is still illegal and hence may not be generalizable to states where it is legal. However, findings from this study provides rich information regarding the barriers and facilitators to recruitment and retention of this population in longitudinal studies in a state with policies similar to Florida.

## Data Availability

The original contributions presented in the study are included in the article/**Supplementary Material**. Further inquiries can be directed to the corresponding author.
