# Supplementary material for: Willingness of pregnant and postpartum women who use marijuana and/or cannabidiol to participate with their offspring in long-term cohort studies: an exploratory study
Source: Front Psychiatry. 2025 Oct 21;16:1641467. doi: 10.3389/fpsyt.2025.1641467 (PMC12583066; doi:10.3389/fpsyt.2025.1641467)
Supplement: Supplementary file 1 [file DataSheet1.docx]

**Focus Group Interview Guide**

**(Questions only)**

**Introduction (10 MINUTES)**

QUESTIONS

1. ***Willingness to Participate in Proposed Cohort Study & Recruitment (10 MINUTES)***

Now, I want to talk about the specific study that we want to conduct.

If you are not currently pregnant, think back to when you were. Try to remember when you were in your second trimester (about months 3 - 6).

- Did you know you were pregnant yet? Do you remember whether you had been to a prenatal visit yet?
- Imagine that when you were pregnant you were asked to join a study that would follow your baby until he or she was about ten years old. The study would gather information about you and your pregnancy and then your baby and his or her development.
- How likely is it that you would be willing to enroll in the study?
- Would it matter who invited you to be in the study?
  - (IF THEY DON’T SAY DOCTOR] Would that change if your doctor invited you to be in the study?
  - [NOT FRIEND. ASK WITH REGARD TO RDS AMONG WOMEN WHO USE MARIJUANA/ CBD] What if a friend or acquaintance told you that she was participating in the study?

1. ***DATA COLLECTION PROCEDURES (20 MINUTES)***
2. ***Pregnant Women***
   - How would knowing that we wanted to collect a lot of personal information about you affect your choice to participate?
   - Would you prefer to share this kind of information with the study team during an interview or by completing a survey?
     - [INTERVIEW] Would you prefer the interview be done in-person or over the phone?
     - [SURVEY] Would you prefer to complete a paper form or complete it online?
   - How would you feel if some of the information collected was sensitive such as divorce or abuse during your childhood or now?
   - How would you feel if we asked about substance use, like tobacco and nicotine, alcohol and, medications your doctors prescribed?
   - How would you feel if we asked about your medical marijuana/ CBD use?
     - How often would you be willing to report the use of marijuana/ CBD?
     - How would you feel most comfortable reporting marijuana/CBD use?
     - Logs or diaries?
     - Short telephone interviews?
     - Links to online surveys sent to your email address?
     - By responding to text messages?

Think about the different ways in which we have discussed collecting sensitive information from women.

- For which method do you think you (or other women) would provide the most honest answers?
- Do you think this differs depending on what substances women are using?
  - What concerns would you have about sharing this information with a study team?

1. ***Biological Specimen Collection Procedures***
   - How willing would you or other women be to be in the study if we asked you to let us look at your medical records?
     - [IF UNWILLING] What concerns do you have about giving us access to medical records?
     - [IF UNWILLING] Are there things that we could do to make you more willing to be in this type of study?
   - How willing would you be to enroll in the study if we did a urine drug screen during your pregnancy?
     - What concerns do you have about routine urine drug screening?
     - [IF UNWILLING] Are there things that we could do to make you more willing to be in this type of study?
   - How willing would you be to enroll in the study if we did a urine drug screen at delivery?
     - What concerns do you have about routine urine drug screening?
     - [IF UNWILLING] Are there things that we could do to make you more willing to be in this type of study?
   - Meconium is the black, tarry stool (or poop) that babies first have after birth. How willing would you be to enroll in the study if we collected meconium to perform a drug (specifically marijuana/ CBD) screen?
     - What concerns do you have about screening your baby’s meconium for marijuana/ CBD or other drugs?
     - [IF UNWILLING] Are there things that we could do to make you more willing to be in this type of study?
   - How willing would you be to enroll in the study if we collected some of the blood from the umbilical cord to perform a drug (specifically marijuana/ CBD) screen?
     - What concerns do you have about screening cord blood for marijuana/ CBD?
     - [IF UNWILLING] Are there things that we could do to make you more willing

An MRI is a way to create a picture of the brain. The procedure does not require any invasive procedures like a needle stick, but it can be uncomfortable for some patients as the MRI can be loud or done in a small space in which you have to remain still. To do these scans on newborn babies, we ask Moms to feed their babies and then we swaddle them for the scan.

- If the study required that we do an MRI scan while you are pregnant, do you think you or other women would agree to be in the study?
  - - - [IF UNWILLING] What concerns would you have about this procedure?
      - [IF UNWILLING] How could we make you feel safe to have the scan while you are pregnant with your child?
- What if we ask you to come once in every trimester for an MRI scan while you are pregnant?
- If the study required that we do an MRI scan on your newborn baby before you went home from the hospital, do you think you or other women would agree to be in the study?
  - - - [IF UNWILLING] What concerns would you have about this procedure?
      - [IF UNWILLING] How could we make you feel safe allowing your newborn to have the scan?
    - What if we asked you to come back to the research center about two weeks after your baby is born to do the MRI scan?
    - What if we asked you to bring your child in for repeat scans every year or two until age five?

Ultrasound is a way to create a picture of the baby during pregnancy. The procedure does not require any invasive procedures like a needle stick, but it can be uncomfortable for some patients as you may have to lie down for approximately one hour.

- If the study required that we do three ultrasounds during your pregnancy, do you think you or other women would agree to be in the study?
  - - - [IF UNWILLING] What concerns would you have about this procedure?

Breastmilk

- - How willing would you be to enroll in the study if we request 1-3 samples of your breastmilk after delivery within the first 6 weeks postpartum?
    - What concerns do you have about providing the breastmilk sample?
    - [IF UNWILLING] Are there things that we could do to make you more willing to provide the breastmilk sample?
  - How willing would you be to enroll in the study if we ask you to provide a sample of colostrum?
    - What concerns do you have about providing colostrum?
    - [IF UNWILLING] Are there things that we could do to make you more willing to be in this type of study?

Developmental assessments look at how your child grows and changes over time. These assessments do not involve any invasive procedures and involve questionnaires or checklists that ask questions about your child’s development over time, including language, movement, thinking, behavior, and emotions. These assessments take place at different stages in your child’s life.

- - [IF UNWILLING] What concerns would you have about developmental assessments for your child at several points over a 5-year period?
  - Would you prefer to share this kind of information with the study team during an in-person interview or by completing a survey?
    - [ IF SURVEY] Would you prefer to complete a paper form or complete it online?

1. ***Child***
   - How would knowing that we wanted to collect a lot of personal information about your child over five years affect your choice to participate?
   - How willing would you be to enroll in the study if we asked you to share sensitive information about your child’s experiences with emotional, physical, or sexual abuse, exposure to domestic violence in the home, substance abuse, mental illness, divorce?
   - Would you prefer to share this kind of information with the study team during an interview or by completing a survey?
     - [IF INTERVIEW] – would you prefer the interview be done in-person or over the phone?
     - [IF IN PERSON] - during a home visit or at a study location?
     - [IF SURVEY] – would you prefer to complete a paper form or complete it online?
     - Would you be willing to allow researchers to come to your home to interview you about it?
2. ***BARRIERS TO ENROLLMENT & RETENTION (15 MINUTES)***

Next, let’s discuss things that might make it difficult for you or someone like you to participate in the study that we have been talking about.

What are some things that would make it hard for you to enroll and stay in the study?

- [IF NO RESPONSES] Prompts
- Lack of time
- Transportation
- Competing demands
- Fear

Are there obstacles related to the study procedures we discussed earlier that we have not already talked about?

- [IF NO RESPONSES] Prompts
- Use of tobacco, nicotine, or alcohol during pregnancy
- Use of prescription or non-prescription medications during pregnancy (e.g., Oxycontin, Percocet methadone, or buprenorphine)
- Use of illicit substance during pregnancy or lactation
- Discomfort with discussing sensitive issues such as violence
- Fear of DCF involvement (e.g. arrest, DCF taking child away) for discussing drug abuse
- Competing demands
- Transportation

Is there anything else that we have not talked about that would keep you or other women like you from enrolling in this type of study?

Is there anything else that we have not talked about that would keep you or other women like you from keeping their child in the study for up to ten years?

1. ***FACILITATORS TO ENROLLMENT & RETENTION (15 MINUTES)***

What could researchers do to make pregnant women more willing to enroll in a study like the one we have been talking about?

1. ***Study Visits***

- Are there things that would make it easier for you to bring your child to study visits?
- Where would be the easiest place to hold study visits?
  - [IF HOME VISITS MENTIONED] Explore home visitation (invasiveness, appropriateness)
  - [IF HOME VISITS NOT MENTIONED] Ask whether the participants would prefer home visits.
- What is the largest number of visits in a year in which your child could participate?

1. ***Maintaining contact and scheduling***

- How would you want us to contact you to schedule visits?
  - When would be the best time to schedule study visits?
- What are ways that researchers can keep in touch with women who have enrolled in this long study?
  - [IF NOT MENTIONED] Explore the usefulness of newsletters, birthday cards, holiday cards

1. ***Incentives***

- If you were to enroll in the study we have been talking about, how would you want us to compensate you for your time and trouble?
- If you were to receive a gift card after each study visit, how much is a fair amount? Should it be a different amount depending on what is done at the visit?
- Is there anything else that would make it easier or make women more willing to enroll in or stay in this study that we have not already talked about?

1. ***PERCEIVED BENEFITS TO PARTICIPATION (15 minutes)***

Do you think there would be any benefit to you or your child for participating in this study?

- [IF YES] Explore these benefits
- [IF NOT MENTIONED] Do you think it would be a benefit to receive additional developmental screenings of your children if you were to participate in this type of study?
- What about the opportunity for early diagnosis and referral to additional health care services and community resources?

What about to women and children in general?

Are there other things that we could do to ensure that being in the study would be a benefit to women?

Are there other things that we could do to ensure that being in the study would be a benefit to children?

1. ***CONCLUSION (5 minutes)***

This brings us to almost the end of our discussion.

- Did we miss something important that you think we should understand about recruiting and retaining pregnant women, moms or other guardians, and their children in long-term studies?
- Do you have any questions?

Thank you so much for providing us with your thoughts about this very important topic. We want to remind everyone to respect each other’s confidentiality and make sure you don’t share information outside of the group. Sharing your knowledge and experiences with us is so valuable to this process, and we appreciate your time.

******************************************************************************
