# Supplementary material for: Willingness of pregnant and postpartum women who use marijuana and/or cannabidiol to participate with their offspring in long-term cohort studies: an exploratory study
Source: Front Psychiatry. 2025 Oct 21;16:1641467. doi: 10.3389/fpsyt.2025.1641467 (PMC12583066; doi:10.3389/fpsyt.2025.1641467)
Supplement: Supplementary file 2 [file Table1.docx]

**Supplementary Material**

| **Table 1**  **Additional quotations from the qualitative analysis** | | |
| --- | --- | --- |
| **Themes** | **Codes** | ***Quotes*** |
| Factors influencing willingness to enroll in a 5- year study | a) Reasons to enroll | *I would enroll in such a study, and I think I would–and I think it would be good to (know about yourself and child)*  *I would...I would also participate…. as long as my child's name wasn't mentioned. (P02)* |
|  | b) Source of invitation to join the study | *It (who invited me) would not affect my decision, as long as I remained anonymous. (P03)*  *I would also like to say it would be more efficient by being referred… because anybody can post, anybody can dress up, or create a flyer or banner…and for me to feel confident if I’m being confronted by somebody that you trust… (P15)* |
|  | c) Impact of personal information collection on enrollment | *As long as my anonymity is kept…remain anonymous. (P07)*  *I'd say no…it will not impact my enrollment decision… only because I trust that my information would be kept private. (P05)*  *It (enrollment) will depend on the information you are asking. (P08)*  *It would depend on what and where the information is going. P (11)*  *I mean, if I am accepting to be enrolled, I would probably be okay because that's part of the study. Otherwise, I wouldn't agree…right?..* *So yeah, I wouldn't have a problem…(P04)* |
| Facilitators and Barriers to Data Collection | a) On personal and sensitive information | *I would prefer through zoom (P06)*  *I would prefer either in-person or through zoom (P09)*  *I wouldn’t want online, for me personally…I prefer in-person (P16)* |
|  | b) On CBD/ Marijuana use | *I wouldn’t use text message, but probably on the phone. (P10)*  *Something on the phone…or I think text or even email.*  *I think the most convenient would be a survey online since some of us may have more than one child, and we're chasing them…(P03)*  *I think that would be good to collect in a survey because they don't necessarily want to talk about (in a focus group) you know, exactly what it was….(P01)*  *On the phone or email or in person. It doesn’t really matter…(P06)*  *If you're collecting information such as demographics and medical history, a Red Cap form is very useful as long as you give a deadline...If you want to collect more subjective information, I'm fine with telephone, in-person, Zoom. But, if it's really that quantitative or "yes or no," or "do you have this?" a survey is very time saving. (P01)* |
|  | c) Consent to review participant’s medical records | *I think it's gonna be a problem… Maybe if you...if you give them something for free. If it involves something that, if somebody's diabetic, can they get some medication for free... Or if they have to do an MRI every other week, every other month…maybe they can have one for free…(P04)*  *I would also like to know or at least have in an I.C.F what exactly would be looked at in the medical records? I will want to know exactly what's being looked at, like the extent of it. And of course, be deidentified and all that stuff. (P03)*  *I'm not opposed to giving my medical information out. It's just the same. I would like to know what it is that you're looking into and at. So, I would feel more comfortable knowing what exactly is being looked through my files from back years and such forth. (P02)* |
|  | d) Consent to collect biological samples | *Urine collection*  *I would have no problem participating in that (collecting urine throughout pregnancy). But I would like to know what the results show because if, for any reason, it's a false positive. …I would like the opportunity to explain or dispute. So, I'm not classified as doing something that I haven't done…otherwise I would have no problem doing that. (P03)*  *Probably not willing, but I guess it would depend on why or what the reason was, and where it’s going? What is it for?(P11)*  Meconium collection  *I'm, of course, totally willing. My only thing is to make sure to have a stipulation within the study design that if the baby has their first meconium stool while in utero then there's that whole other level of NICU care, things like that...(P03)*  *Cord Blood*  *So patients who are looking to do the cord blood donation or cord blood banking may not want to do that because they need for, a STEM cell replacement blood from the umbilical court. So that's just a consideration…(P03)*  *I will be willing to donate cord blood (P10)*  Breastmilk  *If it is a small amount, I would be willing. Because I mostly want to give it to the baby. (P9)*  *I would be willing to do that. And I think other women would be open to that as well. (P10)*  MRI during pregnancy or later for the child  *I’m not familiar with MRIs or if radiation is involved… I want to maybe know the effect on my baby, unborn child, or with, you know, getting [unintelligible] (P13)*  *I would say that they give you a pregnancy test first before they perform any of those MRIs or anything like that, because if you are pregnant, they will not perform, from what I’ve experienced… they will give you an ultrasound…(P15)*  *I think I will have some concern about that, yes. So as a mom, I wouldn't like it, one, because I'm pregnant, and I have baby inside me, and an MRI is not a comfortable thing to have then. And it takes time…* *if I am a mom, and a (marijuana) consumer. And you tell me that the consequences of consumption, and we can do something, if we do it in advance, and we can prevent the consequences then, maybe…If there is no need for an MRI, I don't want to do it (P04)* |
|  | e) Consent to conduct periodical developmental assessment of the child | *I’d be open to that. I wouldn’t have any concerns…. (P10)*  *I would be willing. I’d want that to be anonymous... (P7)*  *I am unsure (P07)*  *I think that if domestic abuse or sexual abuse is happening in the home, they will try to protect their partner and there’s a stigma around that abuse, and so I feel like they may not be willing to do that kind of thing… (P13)*  *I probably would agree, but I would like to know more (P11)* |
| Facilitators to enrollment and retention in the study | a) Incentives | *I think the lactation consultant is a fantastic idea. I think that would be a great incentive, and if the participant said that they're...they don't want it. Then okay, cool…(P03)*  *I think having different incentives for different the length you participate. An MRI is much more stressful than an ultrasound. I feel like the...the time and the stress of the appointment should be a factor in the amount that's given potentially, and or how valuable something is….I do think that there should be kind of a sliding scale based on what it is. And then I think that should be outlined in the very beginning…(P01)*  *For me personally, I was not gonna do it (a research study) if it was a gift card to a certain place….Because the first thing I asked, was “It’s not to a certain place, is it?” because I probably would not be able to get there…and I would like to add __ the needs of the participant how she said for her and her baby, they may need toy at that time or for cash, it might be …so, just a personal need basis…perhaps a gift card.(P15)*  *I feel like in order to keep coming back, maybe the incentives could be switching them up a little bit and not just getting the same thing every single time because they can get kinda bored…(P15)* |
|  | b) Home Visits | *I would allow a researcher to come to my house. Yes, because I am one of them, and I know how difficult this is, and I like the science. So, if you, if you touch the heart of people about how researchers can do better, I'm sure they're going to let you go in the house…(P04)*  *I guess it would depend on that moment how things are…(P14)* |
|  | c) Scheduling the Study Follow up Visits | *I need to be aware to get people around and stuff, like doctor’s appointments, so, I’d maybe want 3 or 4 months in advance…(P15)*  *Like a 6 month…and maybe 2 weeks in advance, like, “Hey, don’t forget, this is your reminder that we’re gonna have our session and, doctor’s appointments, dentist appointments…”(P13)* |
